# Utilization of institutional delivery and associated factors among mothers in Hosanna Town, Hadiya Zone, Southern Ethiopia: A community-based cross-sectional study

**DOI:** 10.1371/journal.pone.0243350

**Published:** 2020-12-03

**Authors:** Demeke Anshebo, Bifitu Geda, Aregash Mecha, Alemu Liru, Ritbano Ahmed

**Affiliations:** 1 Reproductive, Maternal, Neonatal, Child, Adolescent and Youth health-Nutrition, Hadiya Zone Health Department, Hossana, Ethiopia; 2 Department of Nursing, school of Health and Medical Sciences, Madawalabu University, Shashemene, Ethiopia; 3 Department of Public Health Officer, College of Medicine and Health Sciences, Wachemo University, Hossana, Ethiopia; 4 Department of Midwifery, College of Medicine and Health Sciences, Wachemo University, Hossana, Ethiopia; 1. IRCCS Neuromed 2. Doctors with Africa CUAMM, ITALY

## Abstract

**Background:**

Institutional delivery is one of the key interventions that have been proven to reduce maternal and newborn morbidity and mortality. Ethiopia has initiated different efforts to enhance the acceptance of institutional delivery. In spite of this, the number of institutional deliverys is still very low in Ethiopia and varies from region to region. Therefore, this study aimed to assess the utilization of institutional delivery and of factors associated with it among mothers in Hossana Town, Southern Ethiopia.

**Methods:**

This study was a community-based cross-sectional study of mothers who had given birth within 12 months before the study. Data were collected using a pretested questionnaire. During the study period, 403 mothers were selected using the systematic random sampling technique. Data entry was done using EpiData (version 3.1), and data were exported to SPSS (version 24) for analysis. Both bivariate and multivariable logistic regression analyses were used to identify the associated factors at 95% CI.

**Results:**

This study revealed that 53.6% of mothers delivered their infants at health facilities. The factors associated with the institutional delivery were primigravidas (AOR = 3.9; 95% CI, 1.4–4.7), the availability of antenatal care (AOR = 3.4; 95%CI, 1.7–7.2), having planned pregnancies (AOR = 3.9; 95%CI, 1.7–9.3) and the involvement of both parents in decision making (AOR = 2.4; 95%CI, 1.4–2.5). However, when only the mother was involved in the decision making regarding the delivery, the figure decreased by 70% (AOR = 0.3; 95%CI, 0.1–0.8).

**Conclusions:**

The findings of this study indicate that high numbers of births occur without skilled attendants or are non- institutional delivery. In terms of the factors that are associated with institutional delivery, the study suggests that strengthening sustained provision of education during antenatal care and at community levels are crucial.

## Introduction

Throughout the world, roughly three-quarters of all maternal deaths take place during delivery and in the immediate postpartum period [1]. In 2017, approximately 295,000 women died during and directly following pregnancy and childbirth due to inadequate or non-existent care, and 94% of these deaths occurred in the low-resource countries [2]. Ethiopia has significantly reduced its maternal mortality from the 1990s estimate to an average annual reduction rate of 5% or more [3]. However, the levels of maternal mortality in Ethiopia are among the highest in the world. According to the 2016 Ethiopia Demographic and Health Survery (EDHS), 422 women died as a result of complications during pregnancy or childbirth [4]. Most maternal deaths are preventable, as the healthcare solutions to prevent or manage complications are well known [2, 5, 6]. Ensuring that every delivery is attended by a skilled provider is one of the crucial strategies for reducing maternal and newborn morbidity and mortality [6–9].

Institutional delivery refers to childbirth that takes place at medical facilities that are equipped with technology and also under the supervision of skilled birth attendants. It is one of the key interventions that have been proven to reduce maternal and newborn morbidity and mortality [8, 10]. Various medical tools and technologies are used in institutional deliverys to ascertain that the health of the neonates or mothers is not compromised [8, 10]. Delivery in such health facilities has numerous benefits, including increased access to the following: appropriate equipment and supplies that are available on site or through immediate referral to a higher level facility, trained healthcare professionals who provide specific care and attention to newborn babies with special needs to improve their chances of survival and to mothers to reduce the risk of maternal mortality, support for women seeking assistance for delivery at the right time without delaying childbearing, quality care and delivery in an environment that is prepared for emergencies, personnel and equipment to handle emergency circumstances that necessitate immediate medical attention, aid to hasten labour, such as intravenous drips and intramuscular injections during labour, monitoring labour for safe delivery, active management of the third stage of delivery, immediate attention to the newborn, postpartum monitoring and addressing any post-delivery complications in the mother and infant [7, 8, 10].

Global attention to skilled birth attendance has increased remarkably in recent years, although wide gaps in coverage persist across countries [7, 11]. In 2019, 81% of births worldwide were assisted by skilled birth attendants. However, institutional delivery varies from region to region, with figures of 99% in Western Europe, 98% in Eastern and Central Europe, 91% in East Asia and the Pacific, 94% in Latin America and the Caribbean, 94.6% in America and the Caribbean [7] and 57% in Sub-Saharan Africa [7, 11]. Findings from multiple studies conducted in different parts of Ethiopia in the last ten years revealed that the prevalence of institutional delivery is within the range of 12.3% to 78.8% [12, 13].

A number of previous studies have attempted to identify factors associated with institutional delivery in different countries. However, these factors are not the same across different socioeconomic statuses within a society. The socioeconomic factors that have been identified include the following: living in urban settings [12, 14–21], having maternal ages of under 20 [12] and the ages of the mothers being 15 to 24 [22, 23], having high levels of maternal education [15–17, 19–22, 24–32], having high wealth indexes [33, 34], being under 20 years at first marriages and being 20 to 24 years old [13] and having high levels of paternal education [28, 29]. Obstetric factors are as follows: having antenatal care visits during pregnancy [12, 13, 21, 23–26, 35, 36], being primigravida [12, 14, 21, 34, 36, 37], having complications during pregnancy [15,16], having histories of previous institutional delivery [18, 26], having planned pregnancies [37], mothers having adequate knowledge about the danger signs of pregnancy [21, 22] and having birth preparedness plans during pregnancy [17]. Other factors that facilitate the use of institutional delivery include the following: female-headed households [31], women who had made joint decisions with their husbands regarding the place of delivery [31, 37–39], living close to health facilities [36, 39], and staying at a maternity waiting home before delivery [35].

The Government of Ethiopia has developed different strategies to increase the use of institutional deliverys; these include community mobilization by the Health Development Army, service promotion by health extension workers, expansion of health facilities, increasing the availability of supplies and deploying appropriately skilled health professionals. In addition, a number of interventions were implemented to create free home delivery in kebeles [40]. In spite of this, however, the number of institutional delivery is still very low (26%) in Ethiopia. This rate varies across geographic areas, ranging from 97% in Addis Ababa to 15% in Afar [4]. Based on the evidence to date, the findings of multiple studies conducted in the different parts of Ethiopia on similar topics indicate inconsistent coverage, and the various factors [12–20]. Clearly, then, examining the frequencies of institutional delivery and the factors that deter mothers from seeking institutional delivery in specific areas is essential for designing suitable interventions to increase institutional delivery. Therefore, this study attempted to assess the utilisation of institutional delivery and of factors associated with it among mothers in Hossana Town, Southern Ethiopia.

## Methods and materials

This community-based cross-sectional study was conducted from July 1 to 30, 2019 in Hossana Town, Ethiopia. The town is 194 km and 232 km away from Addis Ababa and Hawassa, respectively. The total reproductive age group was 25, 709, and estimated pregnancy was 3,820, which constituted 3.5% of the population. The total reproductive age group were 25, 709, and estimated pregnancy was 3820 in the town which constituted 3.5% of the population. According to the annual report of the town’s health office, the physical health services coverage was estimated to be 100%. The town has one hospital, three health centres and eight urban health extension workers offices, which are all government run. It also has 1 hospital, 35 pharmacies, 22 primary clinics, 19 medium clinics, 2 dental clinics and 2 eye clinics, all of which are private-owned facility [41].

The source population consisted of all mothers who had given birth in Hossana Town within 12 months prior to the study period, and the study population consisted of sampled mothers from this group. Mothers who have lived in the town for at least six months were included, and mothers who were critically ill or cannot able to communicate were excluded.

A sample size of 415 was calculated using a single population proportion formula with the following assumptions, drawn from a study conducted in Wolega: 57% proportion of institutional delivery [24], 95% confidence interval (CI), 5% margin of error and 10% allowance for non-response rate. Systematic sampling was employed, and all kebeles in Hosana Town were included. Before the actual data collection, information on mothers who had given birth within the previous 12 months were compiled from family folders and delivery registration books from each health post in all kebeles to create a sampling frame. Each household was successively given a corresponding house number according to the sampling frame and k-value calculated. If a household had more than one mother with a history of birth in the last 12 months, one of the women was selected using a lottery method. Probability proportional to size allocation was used to allocate study participants in each kebele. Finally, one mother who had given birth in the last 12 months was selected from every sixth household.

For data collection, a pre-tested interviewers administered a questionnaire adapted from instruments used in related studies [12, 13, 15–20, 39, 42, 43] as well as Ethiopian demographic and health survey data [4]. The questionnaire was primarily focused on socio-demographiccharacteristics, obstetric history and participant knowledge related to danger signs during pregnancy and childbirth. A total of 10 midwives, eight with diplomas and two with bachelor’s degrees, who were fluent in the local language were hired for data collection and supervision.

The quality of data was maintained throughout the study. Before data collection began, the questionnaire was translated into Amharic and Hadiyisa and back-translated into English to see the consistency. Additionally, both data collectors and supervisors attended two days of training regarding the study’s objectives, data collection procedures and interview protocol. The questionnaire was also pretested on 5% (21) of the sample size using participants selected from the Fonko town. The completeness, consistency and applicability of the questionnaire were confirmed accordingly. A reliability test, with a Cronbach’s alpha value of 0.72, was conducted on knowledge items related to danger signs of pregnancy and childbirth. During data collection, mothers who were not available upon the data collector’s first visit to their household were revisited two or more times, when necessary, with 2–3 days between visits. Additionally, the investigators and supervisor reviewed filled questionnaires for completeness and consistency of information on a daily basis. Furthermore, prior to analysis, all data were carefully entered and cleaned.

### Measurements

#### Institutional delivery utilization

Giving birth to a child in a health facility under the overall supervision of trained and competent health personnel was classified as: “yes” (mother who gave birth in health facility) or“no”.

#### Knowledge about danger signs during pregnancy and child birth

In this study, danger signs during child birth were assessed using 11 items, with a correct answer was given a score of “1”, and an incorrect answer was given a score of “0”. This questionnaire is scored by calculating the percentage of the total score and categorizing it correspondingly as adequate and inadequate knowledge. **Inadequate knowledge** related to mothers who scored less than 75% of the total score of the knowledge questions, while **adequate knowledge** related to mothers who scored greater than or equal to 75% of the knowledge questions correctly [22, 23].

### Data analysis

Data were checked and entered into EpiData software (version 3.1) before being exported to SPSS (version 24) for analysis. Descriptive statistics were used to summarise the data. Bivariate logistic regression analysis was conducted to identify candidates for multivariate logistic regression analysis. The strength and direction of association between dependent and independent variables were expressed in odds ratio with a 95% CI. To identify independent factors, all independent variables with a *p*-value of ≤ 0.25 in bivariate logistic regression analysis were included in the multivariable logistic regression analysis. Statistical significance was determined using a 95% CI with a p-value of < 0.05. A Hosmer-Lemeshow goodness-of-fit test was used to check that the necessary assumptions for multivariable logistic regressions were fulfilled.

### Ethics approval and consent to participation

Ethical approval was granted by the Research Ethical Review Board of Wachemo University, College of Health Sciences. Moreover, a permission letters were obtained from the Hadiya Zonal Health Department and Hosanna Town health office before data collection began. Finally, written informed consent was obtained from each participant included in the study after its objectives were explained to them. For study participants fewer than 18 years of age, informed consent was obtained from their parent or guardian. Participants were accordingly informed about the purposes, procedures, potential risks and benefits of the study. Confidentiality was maintained throughout the study by excluding personal identifiers, such as names and addresses.

## Results

### Socio-demographic characteristics of the respondents

A total of 403 mothers were interviewed for this study, wherein the response rate was 97.1%. Approximately 83% of the mothers were aged between 20 and 34 years. The majority of the mothers, i.e. 388 (96.3%) were married, 246 (61%) were Hadiya ethnics, 231 (57.3%) were Protestants, and 243 (60.3%) were housewives. In terms of education only 148 (36.7%) had completed secondary or higher education. Other socio-demographic characteristics are shown in Table 1.

**Table 1:** Socio–demographic characteristics of mothers who had given birth in the last 12 months in Hasana Town, November 2019.

Variables	Frequency (N = 403)	Percent
**Age group**		
20–34	334	82.9
35 and above	69	17.1
**Ethnicity**		
Hadiya	246	61.0
Kambata	68	16.9
Amhara	35	8.7
Silte	31	7.7
Gurage	23	5.7
**Religion**		
Protestant	231	57.3
Orthodox	100	24.8
Catholic	40	9.9
Muslims	32	7.5
**Marital status**		
Married	388	96.3
Others*	15	3.7
**Education status of women**		
Cannot read & write	75	18.6
Can read and write	124	30.8
Primary	114	28.3
Secondary & above	90	22.3
**Education status of husband**		
Cannot read & write	59	14.6
Can read and write	83	20.6
Primary	113	28.0
Secondary & above	148	36.7
**Mothers’ occupation**		
Housewives	243	60.3
civil servants	79	19.6
Merchants	81	20.1
**Husband occupation**		
Merchants	148	36.7
Civil servants	140	34.7
Daily laborers	93	23.1
Farmers	22	5.5
**Decision maker at home**		
Husbands	182	45.2
Self	42	10.4
Both	179	44.4

*other include sincle, divorced and widowed

### Obstetric characteristics and prevalence of institutional delivery

Of the 403 study participants, 352 (87.3%) were multigravida. The majority of the mothers, 316 in total (78.4%), had attended antenatal care follow up. Overall, 216 (53%) mothers had delivered their last child at a health facility. Regarding respondents’ knowledge related to danger signs during pregnancy and child birth, 158 (41.1%) had adequate knowledge. The results for other items related to obstetrics and participant knowledge are indicated in Tables 2 and 3.

**Table 2:** Obstetric characteristics of mothers of mother who had given birth in the last 12 months in Hasana Town, November 2019.

Variables	Frequency	Percent
**Gravidity(N = 403)**		
Primi	51	12.7
Multi	352	87.3
**History of institutional delivery(N = 352)**		
No	98	27.8
Yes	254	72.2
**Ever had abortion((N = 352)**		
No	261	74.1
Yes	91	25.9
**ANC follow up((N = 403)**		
No	87	21.6
Yes	316	78.4
**Number of ANC visits((N = 316)**		
< 4 visi	205	64.9
≥4 visits	111	35.1
**Status of last pregnancy (N = 403)**		
Unplanned	117	29.0
Planned	286	71.0
**Institutional delivery (N = 403)**		
No	187	46.4
Yes	216	53.6
**Reasons for non- institutional delivery(N = 187)**		
Lack of transport to health facility	36	8.9
Long distance to health facility	20	5.0
Sudden onset of labour	41	10.2
No respect of health care workers in 1^st^ birth	50	12.4
Poor belief on institution	40	9.9
**Final decision about place of childbirth(N = 403)**		
Both	227	56.3
Husband	103	25.6
Respondent	73	18.1
**Complications of pregnancy**		
No	349	86.6
Yes	58	14.4

**Table 3:** Knowledge on danger signs of mother who had given birth in the last 12 months in Hasana Town, November 2019.

No.	Items	Yes	No
		Frequency (%)	Frequency (%)
1.	Bleeding	359(93.5)	25(6.5)
2.	Severe headache	91(23.7)	293(76.3)
3.	Blurred vision	76(19.8)	308(80.2)
4.	Swollen hands/face	93(24.2)	291(75.8)
5.	High fever	61(15.9)	323(84.1)
6.	Loss of consciousness	102(26.6)	282(73.4)
7.	Difficulty of breathing	51(13.3)	333(86.7)
8.	Severe weakness	62(16.1)	322(83.9)
9.	Severe abdominal pain	96(25.0)	288(75.0)
10.	Accelerated or reduced fetal movement	210(54.7)	174(45.3)
11.	Water breaks	283(73.7)	101(26.3)

### Factors associated with institutional delivery utilization

Under bivariate analysis, women who were mostly associated with an institutional delivery were aged between 20–34, had maternal education of secondary and above level, were primigravida, were not decision makers at home, had a planned pregnancy, and had an ANC during pregnancy. In multivariable logistic regression, women with these conditions were found to be significantly associated with institutional delivery. Primigravida mothers were more likely to give birth at a health institution (AOR = 3.9; 95% CI: 1.4, 4.7). When both parents were involved in determining the place of delivery, the probability of giving birth at a health institution was double or more (AOR = 2.4;95%CI, 1.4, 2.5) than when the decision made only by the husband. In contrast, when the decision was made only by the mother, the probability of institutional delivery was 70% less likely (AOR = 0.3; 95% CI: 0.1, 0.8) as compared to when both parents were involved in the decision making.

In addition, planned pregnancy was increasingly found to be a factor in promoting institutional deliveries (AOR = 33.9, 95% CI: 1.7, 9.3). Furthermore, the likelihood of utilizing institutional delivery increased in mothers who had ANC visits (Table 4).

**Table 4:** Associated factors of institutional delivery mother who had given birth in the last 12 months in Hasana Town, November 2019.

Variables	Institutional delivery(N = 403)		COR(95%CI)	AOR(95%CI)
	No	Yes		
**Age at last pregnancy**				
20–34	139	195	3.2(1.8, 5.6)	1.5(0.8,3.0)
≥35(ref.)	48	21	1	1
**Maternal education status**				
Cannot read & write (ref.)	41	34	1	1
Can read and write	60	64	1.3(.7,2.3)	1.1(.6, 2.2)
Primary	59	55	1.1(.6,2.0)	1.2(.6, 2.3)
Secondary & above	27	63	2.8(1.5,5.3)	1.2(.6, 2.5)
**Gravidity**				
Primi	15	36	2.3(1.2, 4.3)	3.9(1.4, 4.7)**
Multi(ref.)	172	180	1	1
**Decision maker at home**				
Husbands(ref.)	103	79	1	**1**
Self	37	5	0.2(.1, .5)	0.3(.1, .8)*
Both	47	132	3.7(2.3, 5.7)	2.4(1.4, 2.5)**
**Status of last pregnancy**				
Unplanned(ref.)	91	26	1	1
Planned	96	190	6.9(4.2, 11.4)	3.9(1.7, 9.3)**
**ANC followers**				
No(ref.)	73	14	1	**1**
Yes	114	202	9.2(5.0,17.1)	3.4(1.7,7.2)**

Statically significant at **P< 0.01 and *P< 0.05

## Discussions

In this study, the prevalence of institutional delivery was found to be 53.6%, which is higher than that of other studies conducted in Arisi Zone, Dodota, Goba district, Cheha district, Banja, Gondor, Afar, and Wuro-Bunsa, Ethiopia, where utilisation was 12.3%, 18.2, 31%, 47%, 15.7%, 13.8%, and 14.4%, respectively[12, 15, 17, 18, 22–25]. The above-mentioned finding might be due to population differences, the duration of the study, cultural differences, the study setting, sample size or the sampling technique of the present study. In contrast, a lower occurrence of institutional delivery utilisation was observed in this study compared to that of other studies in Bahir-Dar, Woldia, Gambella, Holeta Town and Benishangul-Gumuz, Ethiopia, which were 78.8%, 57%, 63%, 61.6%, and 60.5%, respectively [13, 24, 35, 37, 38]. This variation may be due to differences in the study setting, sociocultural backgrounds or to various interventions carried out in these study areas.

According to the results of this study, the rate of institutional delivery utilisation is lower than in other countries such as Kenya, Zambia, Tanzania, and Ghana where it is reported to be 74%, 77%, 67.3%, and 93.3%% respectively[28, 36, 42, 43]. In contrast, the institutional delivery utilisation was higher than reported in Nepal (51.1%), Pakistan (41.2%), Nigeria (37%), and Eritrea (24.6%) [21, 32, 33, 39]. This finding might be due to the duration of the study, or social, economic or cultural differences.

As revealed by the results of the present study, mothers who had ANC follow-up were reported to have a higher ratio of institutional delivery utilisation. This finding is also supported in studies conducted in Arisi Zone, Bahir-Dar, Nepal, Gondar, Afar, Gambella and Zambia, which reveal that those mothers who had ANC follow-up were more likely to utilise institutional delivery than those who did not have ANC follow-up[12, 13, 21, 23–25, 35, 36]. This might be due to ANC encouraging mothers to deliver at a health facility by providing information on the benefits of institutional delivery, the danger signs during labour, and developing plans to prepare for birth and birth complications. In addition, mothers who had ANC follow-up prepared financially and emotionally for the demands of pregnancy and childbirth.

In this study, primigravida mothers were more likely to utilise institutional delivery, which is in line with what was found in Arisi Zone and Holeta Town(Ethiopia) [12, 37], Nepal [21], Zambia [34], Tanzania [42], and Ghana [43]. The possible explanation for this is that, as the parity increases, a woman’s confidence and experience may increase; as a result, the probability of giving birth at a health facility diminishes for women who have given birth two or more times.

The present study also revealed that those mothers who had discussed the place of delivery with their husbands were more likely to utilise institutional delivery. This finding is similar to studies conducted in Holeta Town and Pawe (Ethiopia) [37, 38], Tanzania [31], and Eretria [39]. In contrast, where only mothers were involved in deciding on the place of delivery, institutional delivery decreased by 70%. A possible reason for this may be the low level of power mothers have in the household.

According to this study, utilisation of institutional delivery was found to be associated with a planned pregnancy. This finding is similar to that of the study conducted in Ethiopia [36]. The possible reason for this finding might be due to the fact that mothers who plan to have a child might want to have a healthy pregnancy and its outcome and thus they might give a great attention for Institutional delivery.

The strengths of this study include the fact that study participants were selected using the probability sampling method to ensure the representativeness of the study, and different approaches were used to maintain the quality of data. As a limitation, this study shares the limitations of a cross-sectional study. Also, this study was not supported by using a qualitative method, therefore, it was not possible to demonstrate detailed reasons from different perspectives for not utilising institutional delivery. Additionally, recall bias could not be ruled out regarding events that took place further from the period of data collection; furthermore, the social desirability bias might be a problem.

## Conclusion

The findings of this study indicate that high numbers of births occur without skilled attendants or are non-institutional deliverys. The factors associated with the utilization of institutional delivery were primigravidas, the availability of antenatal care, having planned pregnancies and the involvement of both parents in decision making. However, when only the mother was involved in the decision making regarding the delivery, the institutional deliveries decreased by 70%. The research finding suggests that strengthening sustained provision of education about pregnancy and delivey during antenatal care and at community levels are crucial. Additionally, empowering women’s participation in the decision-making process within household is essential to inhance institutional delvey.

## Supporting information

S1 File SPSS. (SAV)

S2 File Consent form and questionnaire. (DOC)

## Abbreviations

ANC: Antenatal care
AOR: Adjusted odd ratio
CI: Confidence interval
COR: crude odds ratio
FMOH: Federal ministry of health
SPSS: statistical package for social sciences
WHO: World Health Organisation
