# Supplementary material for: Utilization of institutional delivery and associated factors among mothers in Hosanna Town, Hadiya Zone, Southern Ethiopia: A community-based cross-sectional study
Source: PLoS One. 2020 Dec 3;15(12):e0243350. doi: 10.1371/journal.pone.0243350 (PMC7714344; doi:10.1371/journal.pone.0243350)
Supplement: S2 File — (DOC) [file pone.0243350.s002.doc]

**Annexes 1**

Data collecting tools

**Consent form**

Dear respondents, Good morning/afternoon/evening. My name is _____________. You have been selected to participate in this Post Graduate Research Study on the topic: Institutional delivery utilization and associated factors among mothers in this town. Consequently, the main purpose of this data is only to collect relevant information for this research work.

I would like to ask you a few questions if I may, but you can refuse to answer any question I ask. You may end the interview at any time. Your honest and genuine response to each question has its own value. The data will be used only for academic purposes and the information we collect from you will not be shown to anyone outside of this project.

I approve my agreement to take part in the study as an interviewee with my signature.

Signature _______________________ Date ___________________________

May I proceed with the questions? Yes/No

Name of interviewer__________________ Name of kebele ________________

| **Part I: socio-demographic mothers** | | | | |
| --- | --- | --- | --- | --- |
| Q.# | | **Questions** | **Responses** | **skip** |
| **001** | | Kebele/kifle-ketemas | _____________ |  |
| **002** | | What is your age? | _____________ | In years |
| **003** | | What is your ethnicity? | 1. Hadiya 2. Kambata 3. Silte 4. Gurage 5. Amhara 6. Others__________ |  |
| **004** | | What is your religion? | 1. Orthodox 2. Muslims 3. Protestant 4. Catholic 5. Others specify__________ |  |
| **005** | | What is your marital status? | 1. Married 2. Unmarried 3. Divorced 4. Widowed |  |
| **006** | | What is your educational status? | 1. Cannot read & write 2. Can read and write 3. Primary 4. Secondary & above |  |
| **007** | | What is your husband’s educational status? | 1. Cannot read & write 2. Can read and write 3. Primary 4. Secondary & above |  |
| **008** | | What is your occupation? | 1. House wife 2. Civil servant 3. Merchant 4. Servant 5. Student 6. Daily laborer 7. Other specify_______ |  |
| **009** | | What is your husband’s occupation? | 1. Farmer 2. Civil servant 3. Merchant 4. Student 5. Daily laborer 6. others |  |
| **010** | | Who is the head of the household? | 1. My husband 2. My self 3. My father 4. My mother 5. Other, specify______ |  |
| **011** | | Who is a decision maker at home? | 1. My husband 2. Me 3. Both 4. Other family members |  |
| **Part II: Obstetrics characteristics of mothers** | | | | |
| Q.# | **Question** | | **Response** | **Skip** |
| **014** | How many pregnancies have you ever had? | | 1. Number of gravida____ 2. Number of parity_____ 3. Number of abortion____ 4. Number of live birth___ |  |
| **015** | Last pregnancy | | 1. Planned 2. Unplanned |  |
| **016** | Have you ever attended ANC for last pregnancy? | | 1. Yes 2. No | If “No”, skip to Q. 020 |
| **017** | If yes, number of visits | | ______________ |  |
| **018** | Gestational age at first ANC visit | | ______________ by week |  |
| **019** | Where did you attend ANC visit? | | 1. Health post 2. Health center 3. Hospital 4. Other (specify) _____ |  |
| **020** | Why you didn’t attend? | | 1. I didn’t see any importance of antenatal clinic 2. Long distance to health facility from home. 3. There is service charge 4. No respect of health care workers 5. Other specify_______ |  |
| **021** | Where is the place of first child delivery? | | 1. Home 2. Health post 3. HCs / Hosp. 4. Others___ |  |
| **022** | Was that the place you intended to deliver? | | 1. Yes 2. No |  |
| **024** | Where is the place of last delivery? | | 1. Health institution  2. Home |  |
| **025** | If home, what are the reasons that forced you to give birth at home? | | 1. Lack of transport to health facility 2. Long distance to health facility 3. Sudden onset of labour 4. No respect of health care workers in 1^st^ birth 5. Poor belief oninstitution   Other specify ________ |  |
| **026** | Did mother had any obstetric complication in current pregnancy? | | No  Yes |  |
| **027** | Have you ever heard the term “birth preparedness”? | | No  Yes |  |
| **028** | If yes, what are some things a woman can do to prepare for birth? (multiple answer are possible) | |  |  |
|  | 1. Identify mode of transport 2. Save money 3. . Identify blood donor 4. Identify skilled provider | |  |  |
| **029** | Who made the final decision about place of childbirth? | | 1. Both 2. Husband 3. Respondent 4. Respondent’s father 5. Mother-in-law 6. Father-in-law 7. Other member of family. 8. Health professional 9. 9Other (specify)________ |  |

| **Part III: Knowledge assessment and other factors** | | | |
| --- | --- | --- | --- |
| **038** | Do you know any danger signs which may appear during pregnancy/childbirth? | 1. Yes 0. No | If ‘no’, skip to Q 050 |
|  | If “yes”, what did you know/experience? Multiple answers are possible  1.Bleeding  2. Severe headache  3. Blurred vision  4. Swollen hands/face  5. High fever  6. Loss of consciousness  7. Difficulty breathing  8. Severe weakness  9.Severe abdominal pain  10.Accelerated or reduced fetal movement  11.Water breaks without labor | |  |

**Thank you for your participation!!**
